# Towards a Low-Cost Remote Memory Attestation for the Smart Grid

**DOI:** 10.3390/s150820799

**Published:** 2015-08-21

**Authors:** Xinyu Yang, Xiaofei He, Wei Yu, Jie Lin, Rui Li, Qingyu Yang, Houbing Song

**Affiliations:** 1 Department of Computer Science and Technology, Xi’an Jiaotong University, Xi’an 710049, China; E-Mails: yxyphd@mail.xjtu.edu.cn (X.Y.); hexiaofei@stu.xjtu.edu.cn (X.H.); liruixjtu@stu.xjtu.edu.cn (R.L.); 2 Department of Computer and Information Sciences, Towson University, Towson, MD 21252, USA; E-Mail: wyu@towson.edu; 3 SKLMSE Lab, School of Electronic Information Engineering, Xi’an Jiaotong University, Xi’an 710049, China; E-Mail: yangqingyu@mail.xjtu.edu.cn; 4 Department of Electrical and Computer Engineering, West Virginia University, Montgomery, WV 25136, USA; E-Mail: Houbing.Song@mail.wvu.edu

**Keywords:** smart measurement devices, code injection attack, software-based attestation, smart grid

## Abstract

In the smart grid, measurement devices may be compromised by adversaries, and their operations could be disrupted by attacks. A number of schemes to efficiently and accurately detect these compromised devices remotely have been proposed. Nonetheless, most of the existing schemes detecting compromised devices depend on the incremental response time in the attestation process, which are sensitive to data transmission delay and lead to high computation and network overhead. To address the issue, in this paper, we propose a low-cost remote memory attestation scheme (LRMA), which can efficiently and accurately detect compromised smart meters considering real-time network delay and achieve low computation and network overhead. In LRMA, the impact of real-time network delay on detecting compromised nodes can be eliminated via investigating the time differences reported from relay nodes. Furthermore, the attestation frequency in LRMA is dynamically adjusted with the compromised probability of each node, and then, the total number of attestations could be reduced while low computation and network overhead can be achieved. Through a combination of extensive theoretical analysis and evaluations, our data demonstrate that our proposed scheme can achieve better detection capacity and lower computation and network overhead in comparison to existing schemes.

## 1. Introduction

With the advance of information and network technologies, the Internet of Things (IoT) has revolutionized many conventional areas, including smart home, intelligent transportation and smart grid [1,2,3]. As one of the significant implementations of IoT, the smart grid has attracted growing attention [4]. The smart grid, also denoted as the next generation of the power grid, can provide reliable, secure and efficient energy transmission and distribution [5]. Smart measurement devices (e.g., smart meters), deployed on the user side of the smart grid and applied to measure the power usage information, can not only enhance the interactivity between utilities and customers, but also can increase the efficiency of energy consumption.

Nonetheless, measurement devices (e.g., meters or sensors) can be compromised by cyber attacks (e.g., malicious code injection, *etc.*) launched by adversaries, because they may be connected through computer networks [6]. Using compromised devices, the adversary can launch additional attacks (e.g., injecting false energy demand information) to disrupt the operations of the smart grid (e.g., the dispatch of energy [7], the electricity market [8], *etc.*). As the smart grid consists of a large number of measurement devices, how to detect compromised remote devices efficiently and accurately is a critical issue.

A number of research efforts have been conducted to detect malicious measurement devices through remote software-based attestations [9,10,11,12,13,14,15,16,17]. All aforementioned attestation schemes detecting compromised devices remotely are based on the additional computation time in the verification procedure, which is commonly raised by memory forge attacks [18]. These existing schemes only considered the single remote attestation and ignored the impact of network delay on the detection of remote nodes. In the smart grid, for an easy deployment, smart measurement devices could establish a wireless mesh network to support data transmission. When the smart measurement device is far from the verifier or the network is under malicious attacks (e.g., denial-of-service attacks [19,20], selective forwarding attacks [21,22], *etc.*), the end-to-end delay will increase rapidly and cannot be ignored. To accurately detect compromised devices, existing schemes have to significantly increase the iterations of the verification procedure on remote devices in order to make an increased computation time, which can be distinguished from the data transmission delay. As a consequence of the increased number of verifications, the remote nodes will consume more time on computing the checksum. In addition, when the number of nodes increases, the number of attestations conducted by the verifier will increase, as well. Therefore, the verifier has to generate many attestations for a large system.

In this paper, we propose a low-cost remote memory attestation scheme (LRMA) to verify remote nodes in the advanced metering infrastructure (AMI) network in the smart grid. LRMA takes into account the impact of real-time network delay in the remote attestation and achieves a better attestation performance with a lower overhead. Our approach is an enhanced software-based attestation scheme based on SWATT (SoftWare-based ATTestation technique) [11]. Particularly, in the “challenge-response” verification process, relay nodes will report time differences between their reception of the challenge and response. With these time differences, the verifier can derive the real-time network delay and achieve a better performance without the impact of network delay. In addition, the number of attestation failures on remote nodes will be considered as an indicator to raise the risk level, which is used to show the probability of being compromised. Then, the verifier will arrange the appropriate number of attestations to remote nodes according to their risk level, so that strong nodes will receive less attestations than weak ones. As such, in contrast with existing schemes, our scheme can reduce both the unnecessary computation overhead on remote nodes and the total number of attestations performed by the verifier.

Through a combination of extensive theoretical analysis and simulation experiments, we evaluated the efficiency and security of our proposed scheme in comparison to the existing scheme using a constant attestation rate, in terms of both the computation overhead on remote nodes and the total number of attestations performed by the verifier. Our data demonstrates that our proposed scheme is more efficient than the existing scheme. For example, our proposed scheme can reach the same efficiency as the existing scheme by using a smaller number of attestations (*i.e.*, about 10% of the existing scheme). In addition, the proposed scheme can significantly reduce the computation overhead in each attestation process, while the attestation efficiency remains the constant.

The remainder of this paper is organized as follows: We present the network and threat models in Section 2. We present our proposed scheme in Section 3. In Section 4, we analyze the efficiency and security properties of our scheme. In Section 5, we show experimental results to validate the performance of our proposed scheme. We review related work in Section 7, and conclude this paper in Section 8, respectively.

## 2. Network Model and Threat Model

In this section, we first present the network model and then describe the threat model.

### 2.1. Network Model

**Figure 1:**
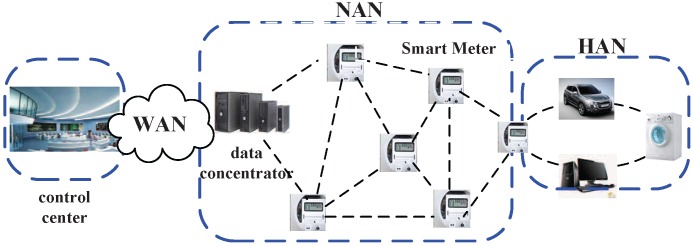
An example of the advanced metering infrastructure (AMI).

Generally speaking, the AMI is a network architecture used to measure, collect, store and analyze the power usage information of consumers and to support the two-way communication between consumers and utilities, leading to an efficient balance between energy demand and supply. Figure 1 illustrates an example of AMI. As we can see, a mesh network is established by smart meters (considered as smart measurement devices), which measure the electricity usage of consumers. The information measured by smart meters will be transmitted to data aggregators/concentrators through the mesh network. The data aggregators then transmit the collected data to utilities through a backbone network (e.g., a wired high-speed network). After that, utilities will perform operations (e.g., billing, demand response and others), leading to effective interactions between consumers and utilities.

**Figure 2:**
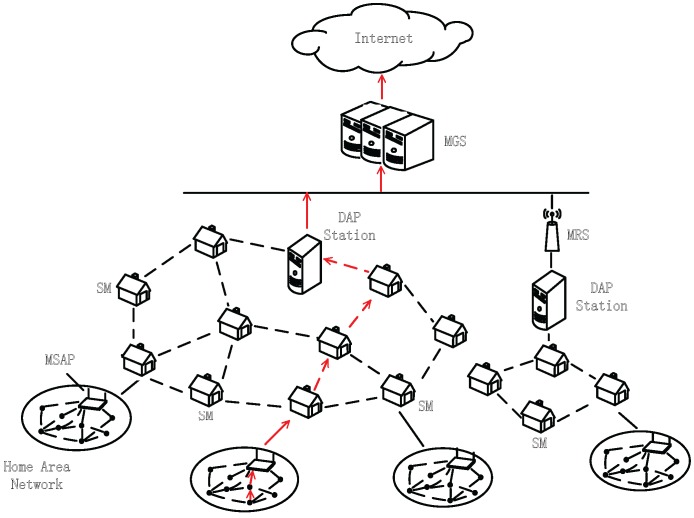
An example of a wireless mesh network for smart grid communication.

We denote each smart meter as a node in the network, and all nodes establish a wireless mesh network, which will be used to transmit data, as shown in Figure 2. There are four types of nodes in the wireless mesh network [23]: (i) the master gateway station (MGS); this represents the AMI head-end connected to the wide area network (WAN), such as the Internet; (ii) DAP station (data aggregation point); this represents the gateway of each subnetwork; (iii) the mesh relay station (MRS); this represents the relay node between the MGS and the DAP station; and (iv) the mesh-station with access point MSAP); this represents a residential smart meter. To obtain accurate data, the DAP-station will verify the memory of all nodes in the network. If the injected malicious code on the remote device is found, the remote code update mechanism will be activated to reset the remote device.

### 2.2. Threat Model

In this paper, we assume that malicious adversaries are intelligent and tend to launch attacks against some nodes with great value. Without loss of generality, we assume that there are multiple individual adversaries in the system, and all attacks are independent repeated trials. Therefore, the attack interval follows an exponential distribution $t_{interval}∼E\left(\lambda \right)$, where λ is the number of attacks in a unit of time.

We also assume that the malicious code injected by the adversary does not exist in the memory of remote devices persistently. To avoid detection, the adversary may eliminate malicious codes after an attack is complete. Therefore, the lifetime of malicious codes on the remote device may not be long enough to be detected by attestation schemes. Furthermore, after the malicious device is detected, it requires some time to find the cause of faults or bugs and to update the hardware or software of compromised nodes. We also assume that the remote node has a high probability in which undiscovered bugs still exist and could be compromised again even after its software is patched.

In addition, because our proposed scheme is an enhanced software-based attestation scheme based on SWATT [11], we have similar assumptions: (i) the verifier is well protected, and information stored in it will not be leaked to the adversary; (ii) the verifier has the knowledge of the hardware structure and software information on the remote devices, including the CPU clock speed, the memory architecture, the instruction set architecture (ISA) of the microcontroller and others; and (iii) the verifier has a local copy of the memory contents of remote devices, so that it can compute the correct checksum, as well.

Because smart measurement devices are often connected through wireless networks, the adversary may launch cyber attacks and compromise measurement devices. For example, the adversary can manipulate the memory contents on compromised devices and launch false data injection attacks [8,24,25,26]. We assume that the adversary will not be able to manipulate the hardware, because it is extremely difficult to replace devices while keeping the system running. Therefore, all assumptions made here are feasible and can be applied to real-world practice.

## 3. Our Approach

In this section, we first present the design rationale of our approach and then show the two main components: dynamic attestation based on the node’s risk level and delay-resilient remote memory attestation.

### 3.1. Design Rationale

**Figure 3:**
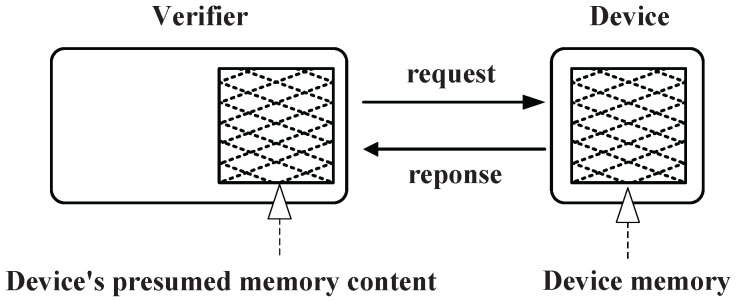
The “challenge-response” protocol [11].

In the existing software-based attestation schemes, the verifier will verify the memory of remote nodes through a “challenge-response” protocol. As shown in Figure 3, the verifier sends a challenge request to the remote device and activates the verification procedure [11]. When the verification procedure generates a checksum based on the contents of its memory, the remote device will send it back to the verifier. Then, the verifier can compute a checksum itself and compare them. If they are not the same or the remote device does not respond to the challenge in the predicted time, the verifier will confirm that the remote device is very likely compromised. Nonetheless, existing attestation schemes do not take into account the data transmission delay and the size of the network. In an AMI, nodes could establish a wireless mesh network, in which the delay cannot be ignored when the remote node is far from the verifier. In addition, the verifier should verify all nodes in the network, but there is no existing scheme that considers the attestation sequence of all nodes.

In this paper, we proposed a low-cost remote memory attestation (LRMA) scheme, which considers the impact of the transmission delay over the network and adjusts the attestation frequency dynamically according to the compromised probability of remote nodes. Consequently, our proposed scheme can reduce the computation overhead on remote devices and the total number of attestations conducted by the verifier. Therefore, our proposed scheme can achieve better performance than existing schemes.

The basic idea of our scheme is described as follows. First, LRMA records the number of attestation failures in the recent $n_{r}$ time slots. The number of failures is used to determine the risk of the node and then to adjust the attestation frequency dynamically. A high risk indicates a high probability that the node is compromised immediately. Then, the verifier adaptively uses a high frequency to attest nodes that shows a high risk and uses a low rate to attest nodes that shows a low risk. In this way, the total number of attestations can be significantly reduced. Second, LRMA estimates the average single-hop delay through reports sent by relay nodes in the route, and then, the verifier can eliminate the impact of the delay when it determines whether the remote node is compromised. To make an accurate detection, the verifier confirms compromised nodes by using the Bayesian classifier when it compares the computation time of the checksum.

LRMA mainly includes two key components: (i) dynamic attestation based on the node’s risk level is used to arrange the sequence of attestations for each node; and (ii) delay-resilient remote memory attestation is used to eliminate the impact of the network delay by deriving the single-hop delay on the path, so that the verifier can confirm compromised nodes with a low computation overhead on remote devices. The notations used in this paper are shown in Table 1.

**Table 1:** Notation.

$v_{i}$	The value of node *i*
$p_{i}$	The probability of node *i* being compromised in an attack
λ	The number of attacks in a unit of time
$n_{r}$	The number of periods to compute the risk value
$R_{i}$	The risk value of node *i*
*p*	The current period
$F_{i,p}$	The number of attestation failure of node *i* in period *p*
*N*	The total number of nodes in the network
*n*	The number of hops in a path
*T*	The unit of time
$T_{v}$	The single verification time
β	The scaling factor for an attestation interval
$n_{v,i}$	The number of verification in a time duration *T*
$d_{\max}$	The maximum end-to-end delay in the network
$\bar{d}$	The average one-hop delay in the network

### 3.2. Dynamic Attestation Based on the Node’s Risk Level

In LRMA, the verifier adopts an adaptive attestation scheme to adjust the rate for conducting attestations based on the risk level on each node.

**Definition 1.** *The risk level of each node is defined as the sum of its attestation failures in recent* $n_{r}$ *slots*.

According to Definition 1, the risk value of node *i* at the *p*-th slot is defined as follows:

(1) $$R_{i}=\left\{\begin{array}{c} \sum_{i=1}^{n_{r}}F_{i,p-i+1}\;\left(p>n_{r}\right)\\ \sum_{i=1}^{p}F_{i,p-i+1}\;\left(p\leq n_{r}\right) \end{array}\right.$$

where $F_{i,p}$ is the number of attestation failures on node *i* at the *p*-th slot.

In our scheme, the risk value of each node is evaluated by a controller. Every attestation failure will lead to an increased risk value. A larger risk value for nodes means a higher probability that nodes are compromised. The risk value of each node will be dynamically updated based on the freshly-updated information.

The attestation frequency performed by the verifier depends on the risk value of nodes. The node with a higher risk value (*i.e.*, the node has a higher probability of being compromised) will have a higher rate to be attested, while the node with a lower risk value will have a lower rate to be attested. In this way, the total number of attestations can be reduced. Determining the rate for carrying out the attestations on remote nodes consists of the following steps:

- Step 1: initialization: Before the network is deployed, the verifier will initialize the risk value on each node. The risk value on each node is set to zero before the first attestation (*i.e.*, the node has not been compromised). Therefore, we have:
  (2) $$R_{i}=0,\;i=1,\cdots ,N$$
  where $R_{i}$ means the risk value of node *i*.
- Step 2: attestation interval generation: The verifier validates nodes with different attestation rates, which are determined by the risk values of the nodes. It is worth noting that the attestation rate is proportional to the security risk of nodes. Therefore, nodes with a high risk value will be verified more frequently by using a higher rate. Then, the *p*-th attestation interval on node *i* can be represented as follows:
  (3) $$T_{i}^{p}=\frac{R_{i}^{p}+\varphi }{{\bar{R}}^{p}+\varphi }\cdot T\cdot \beta \;i=1,\cdots ,N$$
  where *T* is the unit time, *N* is the number of nodes in the network, $R_{i}^{p}$ is the risk value of node *i* at the *p*-th attestation interval, ${\bar{R}}^{p}$ is the average risk value of all nodes at the *p*-th attestation interval, β is the scaling factor for the attestation interval and *φ* is a constant that controls the updated step or convergence speed of the attestation interval $T_{i}^{p}$.
  Then, the attestation time is selected randomly in the *p*-th interval by,
  (4) $$t_{i}=\sum_{j=1}^{p-1}T_{i}^{j}+t_{random},\;t_{random}\in \left[0,T_{i}^{p}\right]$$
  where $t_{random}$ is the random time in period $T_{i}$ and $T_{i}^{p}$ is the *p*-th attestation interval on node *i*.
- Step 3: update: After each attestation process, the verifier will update the risk value of the node based on the attestation result. If the attestation fails, the counter of attestation failures $F_{i,p}$ will increase by one, as well as the risk value $R_{i}$. After a time slot, the risk value will be updated, as well. The risk value will be updated based on summarizing the counters of attestation failures in recent $n_{r}$ slots.

**Algorithm 1** The verifier process (Part I).1:**procedure** ChallengeGeneration($R_{i}$, *φ*, β)2:  **for all**
i∈[1,N]
**do**3:    Generate attestation interval $T_{i}^{p}=\frac{R_{i}^{p}+\varphi }{{\bar{R}}^{p}+\varphi }\cdot T\cdot \beta$4:    Generate attestation time $t_{i}=\sum_{j=1}^{p-1}T_{i}^{j}+t_{random},\;t_{random}\in \left[0,T_{i}^{p}\right]$5:    
$Challenge:key=hash(T_{Verifier})$6:    Attached with RC4 seed $m=\{key∥W_{k}\}$7:    Send challenge message $Verifier\to n:request=\{m∥MAC_{n}\left(m\right)\}$8:    Record sent time $Ts_{Verifier}=Tcurrent_{Verifier}$9:  **end for**10:**end procedure**

**Algorithm 2** The verifier process (Part II).11:**procedure** ChecksumVerification(response, $\Delta T_{i}$, $Ts_{Verifier}$)12: Record received time $Tr_{Verifier}=Tcurrent_{Verifier}$13: 
**if**
$MAC_{n}\left(C\right)=MAC_{n}^{\prime }\left(C\right)$
**then**14:  Generate another checksum $C^{\prime }=\{C_{1}^{\prime }∥C_{2}^{\prime }∥\cdots ∥C_{k}^{\prime }\}$15:  
**if**
$C=C^{\prime }$
**then**16:   
$\Delta T_{Verifier}=Tr_{Verifier}-Ts_{Verifier}$17:   Delay on each hop $\left\{\begin{array}{c} D_{1}=\frac{\Delta T_{1}-\Delta T_{Verifier}}{2},\\ D_{i}=\frac{\Delta T_{i}-\Delta T_{i-1}}{2}\;\left(1<i\leq n-1\right) \end{array}\right.$18:   Get average delay $\bar{d}=\frac{1}{n^{\prime }}\sum_{i=1}^{n^{\prime }}D_{i},\;\left(|D_{i}-\bar{D}|\leq 3s\right)$19:   $T_{checksum}^{\prime }=\Delta T_{Verifier}-n\cdot \bar{d}$20:   Bayesian classifier decision $R\left(c|x\right)=\min_{i=1,2}R\left(c_{i}|x\right)$21:  **else**22:   Checksum Inconsistency Error MAC Inconsistency Error23:  **end if**24: **else**25:  MAC Inconsistency Error26: **end if**27:**end procedure**

**Algorithm 3** The relay node process.1:**procedure** RelayNodeForwarding(*ReceivedPacket*)2: **if**
*ReceivedPacket* = *request*
**then**3:  Forward packet i→i+1:request4:  Record forwarding time $Ts_{i}=Tcurrent_{i}$5: **else**6:  Record forwarding time $Tr_{i}=Tcurrent_{i}$7:  Forward packet i→i-1:response8:  Get time difference $\Delta T_{i}=Tr_{i}-Ts_{i}$9:  Generate report message $m=\{\Delta T_{i}∥MAC_{i}\left(\Delta T_{i}\right)$10: **end if**11:**end procedure**

**Algorithm 4** The remote device checksum generation process.1:**procedure** RemoteDeviceChecksum(key, *W*)2: Set j=13: **while**
j≤k
**do**4:  $A_{j}=RC4\left(key,W_{j}\right)$5:  $C_{j}\leftarrow Mem\left[A_{j}\right]⊕C_{j-1}+RC4_{j}$6:  $C_{j}\leftarrow \;rotate\;left\;one\;bit\left(C_{j-1}\right)$7:  j←j+18: **end while**9: Generate checksum $C=\{C_{1}∥C_{2}∥\cdots ∥C_{k}\}$10: Generate and send response message $response=\{C∥MAC_{n}\left(C\right)\}$11:**end procedure**

### 3.3. Delay-Resilient Remote Memory Attestation

When the remote device is far away from the verifier, the end-to-end delay will be significantly large. This makes the verifier have to increase the iterations of the verification procedure in order to make an accurate decision. In order to eliminate the impact of network delay, we develop a delay-resilient remote memory attestation scheme, which can evaluate the real-time network delay by using the time differences reported by relay nodes in the process of the “challenge-response” protocol.

**Figure 4:**
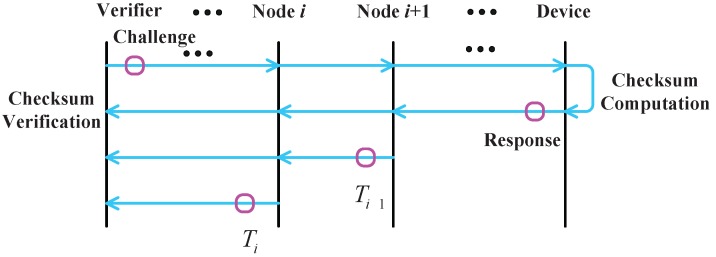
The process of delay-resilient remote memory attestation [27].

As shown in Figure 4, delay-resilient remote memory attestation (DRMA) consists of the following steps:

- Step 1: challenge generation: The verifier sends a random challenge message to the remote device;
- Step 2: challenge transmission: The relay node forwards the challenge message to the destination node and records the time when the challenge message is received by them;
- Step 3: checksum generation: The remote device computes the checksum according to the challenge issued by the verifier and returns the response to the verifier;
- Step 4: checksum transmission: The relay node forwards the checksum from the remote device to the verifier, records the time when the response message is received by the relay nodes and then reports the time difference between the challenge and the response to the verifier;
- Step 5: checksum verification: The verifier separately computes the corresponding checksum and verifies the correctness of the checksum returned by the remote device. If checksums are the same, the verifier will continue to verify the time for computing;
- Step 6: determination: The verifier computes the network delay using the time differences reported by relay nodes and compares the response time with the normal one. In this way, the remote device can determine whether it is compromised or not. DRMA obtains the computing time of the remote device with the consideration of the network delay, in order to achieve greater accuracy for the detection.

In the following subsections, we describe those steps in detail.

#### 3.3.1. Step 1: Challenge Generation

The challenge request will be sent from the verifier to the remote device in order to verify whether the memory is changed by the adversary or not. To avoid an adversary launching replay attacks to a response within a time duration (*i.e.*, returning the old response message to the current challenge), a random challenge message is required. Therefore, the challenge message generation consists of the following three steps:

- Step 1.1: The verifier generates a random key based on the current time of the verifier, which is shown as follows:
  (5) $$Challenge:key=hash(T_{Verifier})$$
  where $T_{Verifier}$ is the current time of the verifier.
- Step 1.2: The message is generated and transmitted:
  (6) m={key∥W}
  where $W=\{W_{1}∥\cdots ∥W_{k}\}$ is the seed for the RC4 function.
- Step 1.3: The verifier sends a challenge message to the remote device with the corresponding message authentication code $MAC_{n}\left(m\right)$ as:
  (7) $$Verifier\to n:request=\{m∥MAC_{n}\left(m\right)\}$$
  where *m* is the message sent by the verifier.
  The verifier records the message when the packet is sent as:
  (8) $$Ts_{Verifier}=Tcurrent_{Verifier}$$
  where $Tcurrent_{Verifier}$ is the current time of the verifier.

#### 3.3.2. Step 2: Challenge Transmission

The challenge transmission of relay nodes consists of the following two steps:

- Step 2.1: The relay node forwards the challenge to the next hop.
  (9) i→i+1:request
  where request is the challenge forwarded by the relay node *i*.
- Step 2.2: The relay node records the time when the request is forwarded.
  (10) $$Ts_{i}=Tcurrent_{i}$$
  where $Tcurrent_{i}$ is the current time of node *i*.

#### 3.3.3. Step 3: Checksum Generation

The remote device computes the checksum in the following steps:

- Step 3.1: The remote device obtains the key from the verifier and initiates the RC4 to generate the random memory addresses as follows:
  (11) $$A_{j}=RC4\left(key,W_{j}\right)$$
  where key is the random key generated by the verifier and $W_{j}$ is the seed for RC4 function.
- Step 3.2: The remote device obtains the memory contents of the addresses to compute the checksum:
  (12) $$C_{j}\leftarrow Mem\left[A_{j}\right]⊕C_{j-1}+RC4_{j}$$
  (13) $$C_{j}\leftarrow \;rotate\;left\;one\;bit\left(C_{j-1}\right)$$
  where $A_{j}$ is the address of the memory that is accessed and $C_{j}$ is the *j*-th checksum.
- Step 3.3: The remote device generates the checksum and returns it to the verifier with the corresponding $MAC_{n}\left(C\right)$:
  (14) $$C=\{C_{1}∥C_{2}∥\cdots ∥C_{k}\}$$
  (15) $$response=\{C∥MAC_{n}\left(C\right)\}$$
  where $C_{j}$ is the *j*-th checksum.

#### 3.3.4. Step 4: Checksum Transmission

The checksum transmission on relay nodes involves the following steps:

- Step 4.1: The relay node records the time when the response is received.
  (16) $$Tr_{i}=Tcurrent_{i}$$
  where $Tcurrent_{i}$ is the current time of relay node *i*.
- Step 4.2: The relay forwards the response to the next hop.
  (17) i→i-1:response
  where response is the checksum forwarded by the relay node *i*.
- Step 4.3: The relay node computes the time difference between the request and the response.
  (18) $$\Delta T_{i}=Tr_{i}-Ts_{i}$$
  where $Ts_{i}$ is the time when the node *i* forwarded the challenge request.
- Step 4.4: The relay node reports the time difference to the verifier after a while.
  (19) $$m=\{\Delta T_{i}∥MAC_{i}\left(\Delta T_{i}\right)$$
  where $\Delta T_{i}$ is the time difference between the challenge and the response forwarding.

#### 3.3.5. Step 5: Checksum Verification

The process to verify the response of the remote device is as follows:

- Step 5.1: The verifier records when the response is received.
  (20) $$Tr_{Verifier}=Tcurrent_{Verifier}$$
  where $Tcurrent_{Verifier}$ is the current time of the verifier.
- Step 5.2: The verifier computes the $MAC_{n}^{\prime }\left(C\right)$ separately and compares it to the $MAC_{n}\left(C\right)$ in the response message.
  (21) $$Compare(MAC_{n}\left(C\right),MAC_{n}^{\prime }\left(C\right))$$
  where $MAC_{n}\left(C\right)$ is the MAC of the checksum on the remote device.
- Step 5.3: If the MAC are the same, the verifier computes checksum $C^{\prime }$ separately and records the computing time $T_{checksum}^{\prime }$.
  (22) $$C^{\prime }=\{C_{1}^{\prime }∥C_{2}^{\prime }∥\cdots ∥C_{k}^{\prime }\}$$
  where $C_{k}^{\prime }$ is the *k*-th checksum computed by the verifier.
- Step 5.4: The verifier compares the checksum $C^{\prime }$ with the *C* in the response.
  (23) $$Compare(C,C^{\prime })$$
  where *C* is the checksum generated by the remote device.

#### 3.3.6. Step 6: Determination

The process of the determination consists of the following steps:

- Step 6.1: If checksums are the same, the verifier will compute the time spent in the entire challenge response.
  (24) $$\Delta T_{Verifier}=Tr_{Verifier}-Ts_{Verifier}$$
  where $Tr_{Verifier}$ is the time when the verifier receives the response and $Ts_{Verifier}$ is the time when the verifier sends the challenge request.
- Step 6.2: The verifier receives the reports from the relay nodes and computes the delay on each hop on the path.
  (25) $$\left\{\begin{array}{c} D_{1}=\frac{\Delta T_{1}-\Delta T_{Verifier}}{2},\\ D_{i}=\frac{\Delta T_{i}-\Delta T_{i-1}}{2}\;\left(1<i\leq n-1\right) \end{array}\right.$$
  where $\Delta T_{i}$ is the time difference reported by relay node *i*.
- Step 6.3: After eliminating the exceptional data by the Pauta criterion [28], the average delay in the network can be estimated by the sample mean:
  (26) $$\bar{d}=\frac{1}{n^{\prime }}\sum_{i=1}^{n^{\prime }}D_{i},\;\left(|D_{i}-\bar{D}|\leq 3s\right)$$
  where $n^{\prime }$ is the number of samples after eliminating the exceptional data and $\bar{D}$ is the sample mean.
- Step 6.4: According to the derived transmission delay, the verifier obtains the time spent on computing the checksum as follows:
  (27) $$T_{checksum}^{\prime }=\Delta T_{Verifier}-n\cdot \bar{d}$$
  where $\Delta T_{Verifier}$ is the time difference between the challenge sent and the response received by the verifier.
- Step 6.5: The verifier computes the posterior probability and the conditional risk based on the minimum risk Bayesian classifier [28].
  (28) $$P\left(\omega_{j}|x\right)=\frac{p\left(x|\omega_{j}\right)P\left(\omega_{j}\right)}{\sum_{i=1}^{2}p\left(x|\omega_{i}\right)P\left(\omega_{i}\right)},\;j=1,2$$
  (29) $$R\left(c_{i}|x\right)=\sum_{j=1}^{2},\lambda \left(c_{i},\omega_{j}\right)P\left(\omega_{j}|x\right),\;j=1,2$$
  where the observation *x* is the difference between the received checksum computing time $T_{checksum}$ and the verifier’s computing time $T_{checksum}^{\prime }$. The *a priori* probability $P(\omega_{j})$ is the probability of the node being compromised, which can be determined by the compromised history of the network. The class conditional probability density $p(x|\omega_{j})$ can be estimated by the response time distribution of both the compromised nodes and the normal nodes with the experiment. The decision function $\lambda (c_{i},\omega_{j})$ is determined by the damage caused by the wrong decisions according to the importance of the nodes.
- Step 6.6: Comparing the conditional risk $R(c_{i}|x),i=1,2$, the verifier will find the decision that minimizes the conditional risk, as follows:
  (30) $$R\left(c|x\right)=\min_{i=1,2}R\left(c_{i}|x\right)$$
  where *c* is the minimum risk Bayes decision, *x* is the observation and the $c_{i}$ is the corresponding decision. Then, the verifier can confirm whether the remote node is compromised according to *c*.

## 4. Analysis

In this section, we analyze the performance of our proposed attestation scheme from the following two aspects: efficiency and security.

### 4.1. Efficiency Analysis

To measure the efficiency, we consider the following metrics: (i) the number of detected attacks is defined as the total number of attacks, which have been perceived by the verifier in a given time window; and (ii) verification time is defined as the average time taken for one attestation. Because the original software-based attestation scheme, such as SWATT [11], does not involve the verification frequency on the verifier, we assume that each node will be verified at the same rate.

#### 4.1.1. The Number of Detected Attacks

According to the threat model in Section 2, the attack interval of the adversary has an exponential distribution $t_{interval}∼E\left(\lambda \right)$, where λ is the number of attacks in a time window *T*. Denote the compromised probability of node *i* in a single attack as $p_{i}$. Then, in a time window *T*, the number of attacks against node *i* is $\lambda p_{i}$. The average number of attestations on the node *i* is $n_{v,i}=\frac{T}{T_{v}}$. Then, the probability of the attack perceived by one attestation is $\frac{\lambda p_{i}\cdot t_{a}}{T}$, and the total number of detected attacks in the time window *T* is:

(31) $$n_{d,i}=\frac{\lambda p_{i}\cdot t_{a}}{T}\cdot n_{v,i}=\frac{\lambda p_{i}\cdot t_{a}}{T_{v}}$$

In the original software-based attestation scheme, such as SWATT [11], the total number of detected attacks in the time window *T* is:

(32) $$n_{d,original}=\sum_{i=1}^{N}n_{d,i}=\sum_{i=1}^{N}\frac{\lambda \cdot t_{a}}{NT\beta }=\frac{\lambda \cdot t_{a}}{T\beta }$$

In the LRMA, according to Definition 1, there is:

(33) $$n_{r}\cdot n_{d,i}=R_{i}$$

where $n_{r}$ is the number of periods to compute the risk value and $R_{i}$ is the risk value of node *i*.

Then, we have:

(34) $$T\beta {\left(R_{i}+\varphi \right)}^{2}-T\beta N\left(R_{i}+\varphi \right)-n_{r}\cdot \lambda p_{i}t_{a}\left(\bar{R}+\varphi \right)=0,\;i=1,2,\cdots ,N$$

Let $F\left(p_{i}\right)=R_{i}+\varphi$, and then, we have:

(35) $$F^{\prime }\left(p_{i}\right)=\frac{Nn_{r}\lambda t_{a}\left(\bar{R}+\varphi \right)}{\left(2R+\varphi \right)NT\beta -n_{r}\lambda t_{a}p_{i}}$$

(36) $$F^{\prime \prime }\left(p_{i}\right)=\frac{\left(T\beta NF^{\prime }-n_{r}\lambda t_{a}\right)\cdot 2F^{\prime }}{\left(2R+\varphi \right)NT\beta -n_{r}\lambda t_{a}p_{i}}$$

As we can see, if we have $n_{r}\leq \frac{(2R+\varphi )NT\beta }{\lambda t_{a}p_{i}}$ and $n_{r}\geq \frac{(2R+\varphi -N\bar{R}-N\varphi )NT\beta }{\lambda t_{a}p_{i}}$, we can get $F^{\prime }\left(p_{i}\right)>0$ and $F^{\prime \prime }\left(p_{i}\right)>0$. Therefore, in the LRMA, the total number of detected attacks in the time window *T* is:

(37) $$n_{d,LRMA}=\sum_{i=1}^{N}\frac{R_{i}}{n_{r}}=\frac{1}{N}\sum_{i=1}^{N}F\left(p_{i}\right)\frac{N}{n_{r}}-\frac{N\varphi }{n_{r}}\geq F\left(\frac{1}{N}\sum_{i=1}^{N}p_{i}\right)\frac{N}{n_{r}}-\frac{N\varphi }{n_{r}}=n_{d,original}$$

If and only if $p_{1}=p_{2}=\cdots =p_{N}=\frac{1}{N}$, the equality holds.

#### 4.1.2. Verification Time

The adversary may launch a memory verification attack against the attestation by generating the checksum accurately [11]. This means that the adversary could copy the original memory contents into the empty memory and carry out a check of whether the memory access is one of them. If the memory access touches an altered location, the adversary will load the correct copy instead.

Nonetheless, the inserted if statements in the verification procedure will increase the detection probability. The slowdown is related to the number of memory accesses of the verification procedure. Generally speaking, if the verifier expects to detect this slowdown, the computing time for the checksum should be significantly larger than the worst case network delay in order to make it distinguishable from the network delay. Then, we have:

(38) $$n_{ex}\cdot t_{ex}\gg n\cdot d_{\max}$$

where $n_{ex}$ is the number of iterations in the verification procedure, $t_{ex}$ is the increased time of executing an if statement, *n* is the number of relay nodes in the path and $d_{\max}$ is the maximum delay in a single-hop.

Then, we have:

(39) $$n_{ex}\cdot t_{ex}=n\cdot d_{\max}\cdot \alpha$$

where $d_{\max}$ is the end-to-end delay in the network and α is the scale factor.

According to our proposed attestation scheme, we estimate the one-hop transmission delay $\bar{d}$ on the path in the network. Then, we only need to ensure that:

(40) $$n_{ex}^{\prime }\cdot t_{ex}+n\cdot \bar{d}\gg n\cdot d_{\max}$$

where $n_{ex}^{\prime }$ is the number of iterations in DRMA. Then, we have:

(41) $$n_{ex}^{\prime }=n_{ex}-\frac{n\cdot \bar{d}}{t_{ex}}$$

To summarize, in the case where the checksum computing time is significantly distinguished from the network delay, the cost of our proposed attestation scheme is less than the ones without considering network delay. When the transmission path is long, the average network delay increases, leading to a low overhead of the proposed scheme.

### 4.2. Security Analysis

We now analyze the security of our proposed scheme against several attacks, including: (i) the local forged checksum is defined as the checksum generated by compromised nodes; (ii) the relay nodes’ false reports on the time difference are defined as the false time difference reported by compromised relay nodes; (iii) the relay nodes that deferred forwarding are defined as the reports delayed by the compromised relay nodes deliberately; and (iv) the collusion of relay nodes is defined as the attack that is launched by several compromised nodes collaboratively.

#### 4.2.1. Local Forged Checksum

In this case, the adversary may attempt to use mechanisms to generate the correct checksum. In this paper, the checksum generation program is designed as a series of computation process. Because every computation requires the result of the previous computation, the adversary cannot generate an accurate checksum in a specific time. If the adversary generates the checksum randomly, the probability that the adversary can generate the accurate checksum coincidentally is only $\frac{1}{2^{k}}$ when the checksum has *k* bits. As long as the checksum is long enough, it is hard for the adversary to guess the correct checksum.

#### 4.2.2. Relay Nodes False Reports on the Time Difference

The adversary may attempt to exploit compromised nodes to disrupt the delay estimation process. Nonetheless, the time differences reported by relay nodes should be a sequence, which is listed as follows:

(42) $$\Delta T_{1},\Delta T_{2},\ldots ,\Delta T_{n-1}$$

where $\Delta T_{i}$ is the time difference between the challenge and the response forwarded on the relay node *i*.

Then, the delay between two adjacent nodes is:

(43) $$\left\{\begin{array}{c} D_{1}=\frac{\Delta T_{1}-\Delta T_{Verifier}}{2}\\ D_{i}=\frac{\Delta T_{i}-\Delta T_{i-1}}{2}\;\left(1<i\leq n-1\right) \end{array}\right.$$

According to the central limit theorem, the delay approximates a normal distribution, and we have:

(44) $$D_{i}∼N\left(\mu ,\sigma^{2}\right)$$

where expectation μ is estimated by the sample mean $\bar{D}$ and variance $\sigma^{2}$ is estimated by the sample variance $s^{2}$.

If the relay node *i* sends false time difference data, the time difference between adjacent nodes will significantly deviate from the sample mean. This means that the time difference $D_{i}$ will satisfy the following inequalities:

(45) $$|D_{i}-\bar{D}|>3\sigma ,\;or\;|D_{i+1}-\bar{D}|>3\sigma$$

Therefore, the verifier could remove the false data by the Pauta criterion [28].

#### 4.2.3. Relay Nodes’ Deferred Forwarding

In this case, the adversary may attempt to make compromised relay nodes hold the packet for a period of time before forwarding it. The adversary may also launch the aforementioned attacks, such as reporting the false time differences at the same time. When the relay nodes hold the packet deliberately before forwarding, the next relay node will receive packets a bit later. Nonetheless, compromised nodes cannot manipulate the time difference $\Delta T_{i}$ reported by other normal relay nodes.

Therefore, the transmission delay is still correct, except the delays $D_{i}$ and $D_{i+1}$, which are related to the compromised node *i*. Then, there are only $D_{i}$ and $D_{i+1}$ to deviate from the sample mean, which can be easily detected according to the method described above.

#### 4.2.4. Relay Nodes Collusion

In this case, the adversary may attempt to disrupt the normal delay estimation process, using adjacent compromised nodes to launch a collusion attack, in which the adversary deceives the verifier into mistaking the extra computing time for the network delay. Nonetheless, only the adjacent nodes compromised by the adversary could manipulate the delay $D_{i}$.

For an *n*-hop path, if the adversary takes control of *m* consecutive nodes adjacent to the destination node, the increased delay can be divided into *m* parts, which will be added to the report of each compromised node. In order to avoid being eliminated by the Pauta criterion [28], the time difference reported by compromised nodes should be:

(46) $$\Delta^{\prime }T_{n-m+i}=\Delta T_{n-m+i}-i\cdot \frac{2\alpha T_{if}}{m}\;\left(1\leq i\leq m\right)$$

where the $T_{if}$ is the increased computing time caused by the extra if statements and α is the scale factor.

Furthermore, the manipulated delay between the adjacent nodes is:

(47) $$D_{i}^{\prime }=\frac{\Delta T_{i}^{\prime }-\Delta T_{i-1}^{\prime }}{2}=D_{i}+\frac{\alpha T_{if}}{m}\;\left(n-m+1\leq i\leq n\right)$$

To avoid being regarded as outliers by the verifier, the manipulated network delay should meet the following condition:

(48) $$\begin{array}{cc} \min & m\\ s.t. & \left\{\begin{array}{c} |D_{i}^{\prime }-{\bar{D}}^{\prime }|\leq 3\sigma \;\left(n-m+1\leq i\leq n\right)\\ |D_{i}-\bar{D}|\leq 3\sigma \;\left(1\leq i\leq n-m\right) \end{array}\right. \end{array}$$

where the $\bar{D}$ is the normal sample mean and ${\bar{D}}^{\prime }$ is the sample mean after some reports are changed.

Therefore, we have:

(49) $$m\geq \frac{1}{\frac{3\sigma }{\alpha T_{if}}+\frac{1}{n}}$$

σ is the standard deviation; $T_{if}$ is the increased computing time caused by the extra if statements; and α is the scale factor.

Hence, with the increase of the number of hops *n* in the path, the number of compromised nodes *m* that the adversary should control increases, as well. For example, if n=10, *m* is about five (when σ is 0.001, $T_{if}$ is 0.3 and α is 0.5). Notice that when the remote node is far from the verifier, the adversary needs to control more nodes, and a successful attack becomes harder.

## 5. Performance Evaluation

We carried out experiments and compared the performance of our proposed attestation scheme with one representative attestation scheme [11]. In the following, we first present the evaluation methodology and then show the evaluation results.

### 5.1. Methodology

We implemented our proposed scheme and one existing scheme on NS-3 and conducted performance comparison. As most of the existing attestation schemes do not consider the metrics (e.g., network delay) that we focused on in this paper, we choose a typical software-based attestation scheme [11] (also called SWATT) as a baseline scheme and compare its performance with our proposed scheme. In SWATT, the verifier sends a challenge to the remote device and then determines whether it is compromised based on the response time in the “challenge-response” process.

In our experiments, we consider a scenario of 225 nodes that form a 15×15 array. Nodes are deployed in a 0.750m×0.750m area uniformly, *i.e.*, each node is deployed in a square with a side length of 50m. The verifier, which is located at (0,0), verifies nodes on different locations in the network. With the loss of generality, we assume all nodes are the same and have the 802.11b NICs in ad hoc mode. In our evaluation setup, 802.11b physical layer with DsssRate of 1 Mbps and the AODV route protocol are used.

To simulate a real situation, we assume that the adversary prefers to attack some important nodes in the network and to remove attack traces after the injection of malicious code, in order to prevent the detection from the verifier. To simplify the simulation, we assume that all attacks are independent and that the time of each attack has an exponential distribution with a rate parameter λ, which represents the average number of attacks in a unit of time. We also assume that the life time of malicious codes is 20% of the unit interval, whereas the simulation lasts for 100 unit intervals.

To evaluate the efficiency of our proposed scheme, we consider two cases: (i) attestation efficiency is used to compare the efficiency with the same number of attestations; and (ii) attestation overhead is used to compare the required attestation to reach the same efficiency. We compare our proposed scheme and the baseline scheme in terms of following metrics: (i) successful attestation is defined as the number of attestations that detect an attack; (ii) the number of attestations is defined as the total number of attestations; and (iii) the number of undetected attacks is defined as the number of attacks that are not detected.

In addition, to evaluate the computation overhead on remote nodes, we also consider the overhead of computing the checksum, which tends to compare the overhead of the remote device computing the checksum. To this end, we define the metric response time as the time difference between the challenge request sent and the checksum response received.

### 5.2. Results

We now show the effectiveness of our proposed scheme in terms of the efficiency and overhead of attestations.

#### 5.2.1. Attestation Efficiency

**Figure 5:**
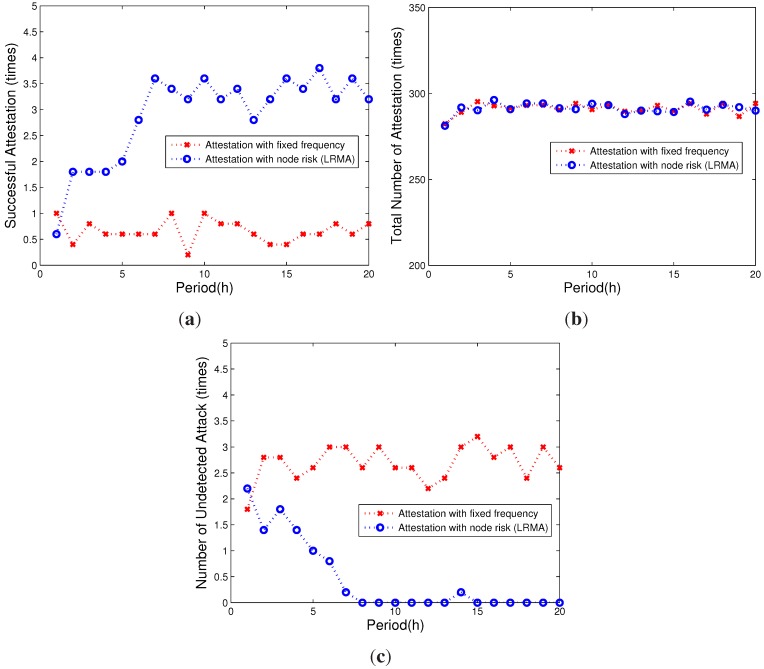
The attestation efficiency comparison. (**a**) Successful attestation; (**b**) number of attestations; (**c**) number of undetected attacks.

Figure 5a,b shows that with the same number of attestations, our scheme achieves a higher efficiency than the baseline scheme. Our scheme can detect more attacks after a time duration by updating the risk value of each node. As we can see from the figure, our proposed scheme can detect all compromised nodes in the network after evaluating risk values of individual nodes and performing the verification. In contrast, the scheme with the fixed attestation rate cannot change the verification interval dynamically, so that the performance remains constant, leading to poor efficiency. As shown in Figure 5c, with the same number of verifications, our proposed scheme is capable of arranging more verification of nodes that have a high risk value.

#### 5.2.2. Attestation Overhead

**Figure 6:**
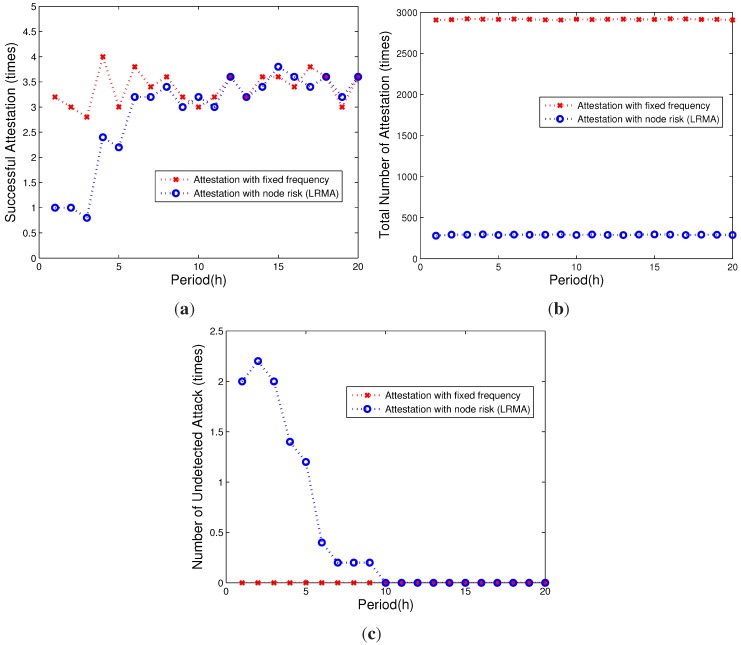
The attestation overhead comparison. (**a**) Successful attestation; (**b**) the number of attestations; (**c**) the number of undetected attacks.

Figure 6a,b shows the overhead of our proposed scheme in comparison to the baseline scheme. To achieve the same efficiency, our scheme needs less attestations. Our scheme only needs around 10% of the attestations in comparison to the baseline scheme. As we can see from the figure, the verification efficiency of our proposed attestation scheme remains the same as the baseline scheme because compromised nodes keep the same attestation, and only reliable nodes get less attestation. Our scheme can detect almost all of the attacks in the network. As shown in Figure 6c, because our proposed attestation scheme is based on the vulnerability risk of nodes, nodes that have a highly probability of being compromised will have a high probability of being verified. For nodes that have a low risk of being compromised, an appropriate verification will be used. In contrast, all nodes will have the same verification when the baseline scheme is used, leading to a higher attestation overhead. From the figures, we observe that our proposed attestation scheme achieves the same efficiency with only 10% verification overhead in comparison to the baseline scheme.

#### 5.2.3. Overhead of Computing the Checksum

We also evaluate the response time *versus* the number of hops. We consider two cases: (i) low overhead means that remote devices take only a little time for the computing time; and (ii) high overhead means that remote devices take much time for an obvious computing of the time against the network delay.

**Figure 7:**
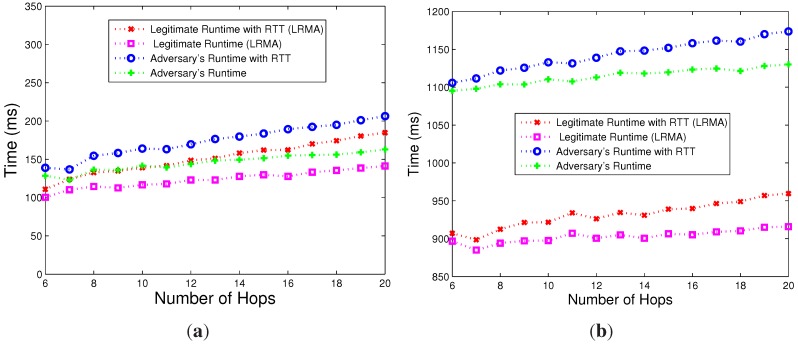
The response time *vs*. the number of hops. (**a**) Low overhead; (**b**) high overhead.

Figure 7a shows the results for the first scenario. As we can see, with the increase of the number of hops, the verification time that a verifier takes gradually increases. The network delay of normal nodes is larger than the extra computing time of compromised nodes when the number of hops reaches 12. Therefore, it is difficult for a verifier to distinguish the extra computing time caused by an attack from the network delay.

Figure 7b shows the response time *versus* the number of hops for the second scenario. As we can see, the increased number of memory accesses makes the extra computing time of the compromised nodes significantly lager than the network delay. Nonetheless, the total overhead is uncommonly lager, as well.

Therefore, our proposed scheme can distinguish the extra computing time of compromised nodes from the network delay with a very low overhead.

## 6. Discussion

In the following, we discuss several issues: the attack interval, a hybrid network and the capability of extensibility.

- Attack interval: In this paper, we assume that all attacks are independent and that the attack interval has an exponential distribution with a rate parameter λ. In a realistic environment, there may be more than one adversary in the network, and the attack interval may not be in a regular pattern. The performance in this case shall be analyzed further, and the proposed scheme shall be enhanced to address this issue.
- Hybrid network: In this paper, we consider that the network is uniform and that all of the nodes in the network are the same. Nonetheless, the realistic AMI may be a hybrid network. As such, the network delay of remote nodes in different locations may not be alike, as well as the computation time of the checksum. Then, the verifier must have a proper mechanism to distinguish them and confirm compromised nodes in the network. Therefore, how to deal with the hybrid network is another issue for future study.
- Extensibility: In addition to the smart grid, there are numerous systems where the legacy system exists. Our proposed scheme is generic and can be extended to systems where the remote memory attestation is needed, but the hardware or computing capability is limited. As shown in our analytical and evaluation results, our proposed scheme can achieve a great performance with a lower overhead.

## 7. Related Work

In this section, we conducted a literature review in the area of remote memory attestation, which is closely related to our study. In a trusted computing environment, remote attestation generally requires specific hardware, which is commonly called the trusted platform module (TPM) [29]. Nonetheless, remote attestation schemes based on the TPM [30] cannot be directly applied to the smart grid because of the limitation of hardware and software on specific devices deployed in the smart grid. To avoid the limitation of the hardware, software-based attestation schemes have been developed for legacy systems. For example, Seshadri *et al.* proposed a software-based attestation for embedded devices called SWATT [11]. In SWATT, the verifier sends a challenge request to remote devices, which activates the verification procedure on remote devices with a generated checksum. Then, remote devices respond to the checksum of the verifier. Notice that if the adversary launches the memory verification attack that attempts to compute the correct checksum, the verifier can still detect the slowdown raised by the extra if statements. Seshadri *et al.* also proposed FIRE (Forgery-resilient Intrusion detection, Recovery, and Establishments of keys) [31], SCUBA (Secure Code Update By Attestation) [9] and SAKE (Software Attestation for Key Establishment) [32], which all use indisputable code execution (ICE) to verify the correction of memory in remote devices based on the response time of remote devices. In addition, Seshadri *et al.* proposed a verification called Pioneer [33], which verifies the code integrity and enforces untampered code execution on an untrusted legacy host.

Song *et al.* proposed a one-way memory attestation protocol (OMAP) for smart meters [10], which uses built-in values as challenges to compute the checksum. Although OMAP does not need the challenge sent from the verifier, it may be subject to the memory replication attack. Haemin Park *et al.* proposed a software-based attestation technique, namely SMATT [34], to address issues, including the verification overhead for large-scale networks and the evasion of attestation by the memory replication. Nonetheless, SMATT requires a public key infrastructure (PKI), which is considered as the basis for the secure communication between smart meters and verifiers. Nonetheless, using the public key in the smart grid is still questionable due to the hardware and software deployment in the smart grid, which consists of a number of heterogeneous devices. Micheal LeMay proposed an attestation scheme, namely CAK (cumulative attestation kernel) [35], which addresses the inconsistencies of time-of-use-to-time-of-check (TOUTTOC). CAK is a cumulative attestation for embedded systems to prevent the attack in the verification process. It requires asymmetric key pairs for devices, which limit its use in the smart grid.

In this paper, we proposed a low-cost remote memory attestation for the smart grid. Different from existing attestation schemes, our proposed attestation scheme takes into account the impacts of the network delay on the detection of remote nodes and does not require key management and specific hardware or software support. Therefore, our proposed scheme can be useful in the smart grid that consists of numerous legacy devices.

## 8. Conclusions

In this paper, we presented a low-cost remote memory attestation scheme (LRMA), which achieves a low overhead in the process of verification. LRMA considers the number of attestation failures as the risk of each node and adjusts the attestation frequency of remote nodes according to their risk levels. In addition, LRMA eliminates the impact of the real-time transmission delay by investigating the time differences reported by relay nodes. Using the risk level of each node and the real-time delay in the network, the verifier could detect compromised nodes with a low computation and network overhead. Through a combination of theoretical analysis and experiments, we demonstrated that our scheme could achieve a higher efficiency of detection by the lower verification overhead than the existing remote software-based memory attestation scheme.
